# Is Disrupted Mitophagy a Central Player to Parkinson’s Disease Pathology?

**DOI:** 10.7759/cureus.35458

**Published:** 2023-02-25

**Authors:** Tsz Ki Ko, Denise Jia Yun Tan

**Affiliations:** 1 Otolaryngology, College of Life Sciences, Leicester Medical School, George Davies Centre, Leicester, GBR; 2 ENT, Nottingham University Hospital, Nottingham, GBR

**Keywords:** parkinson's disease, lewy body, parkin, pink1, mitophagy

## Abstract

Whilst the pathophysiology at a cellular level has been defined, the cause of Parkinson’s disease (PD) remains poorly understood. This neurodegenerative disorder is associated with impaired dopamine transmission in the substantia nigra, and protein accumulations known as Lewy bodies are visible in affected neurons. Cell culture models of PD have indicated impaired mitochondrial function, so the focus of this paper is on the quality control processes involved in and around mitochondria.

Mitochondrial autophagy (mitophagy) is the process through which defective mitochondria are removed from the cell by internalisation into autophagosomes which fuse with a lysosome. This process involves many proteins, notably including PINK1 and parkin, both of which are known to be coded on genes associated with PD. Normally in healthy individuals, PINK1 associates with the outer mitochondrial membrane, which then recruits parkin, activating it to attach ubiquitin proteins to the mitochondrial membrane. PINK1, parkin, and ubiquitin cooperate to form a positive feedback system which accelerates the deposition of ubiquitin on dysfunctional mitochondria, resulting in mitophagy. However, in hereditary PD, the genes encoding PINK1 and parkin are mutated, resulting in proteins that are less efficient at removing poorly performing mitochondria, leaving cells more vulnerable to oxidative stress and ubiquitinated inclusion bodies, such as Lewy bodies. Current research that looks into the connection between mitophagy and PD is promising, already yielding potentially therapeutic compounds; until now, pharmacological support for the mitophagy process has not been part of the therapeutic arsenal. Continued research in this area is warranted.

## Introduction and background

Parkinson’s disease and genetics

**Figure 1:**
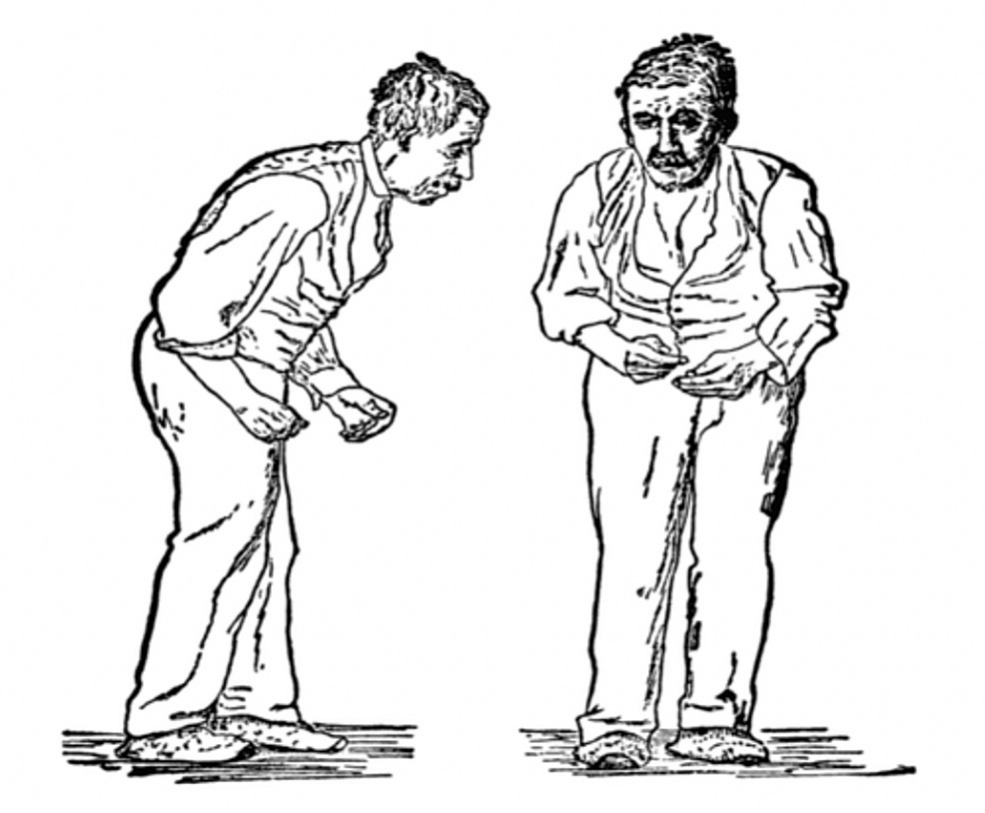
Figure showing Parkinson’s disease patient from the front and from the side. His face is expressionless, his posture is stooped forward, and he has difficulty walking. Figure taken from [5], and permission was obtained from the original publisher to reproduce the content.

Parkinson’s disease (PD) is the second most common neurodegenerative disorder following Alzheimer’s, affecting 1% of adults over the age of 60. It is characterised by dopaminergic cell loss in substantia nigra pars compacta of the midbrain, and patients are presented with the classic symptoms of parkinsonism: bradykinesia, rigidity, resting tremor and postural instability (Figure 1) [1]. PD is a universal disorder present across the world, with a crude incidence rate of 5-19 people per 100,000 populations per year and a prevalence of 70-250 people per 100,000 populations, which rises with the increase in age [2,3]. In addition to placing enormous physical strain on the patients, PD also places a financial strain on the health service. Parkinson’s care costs the National Health Service (NHS) over £220 million per year and annual care for patients with advanced Parkinson’s (>75% of waking time in a state of decreased mobility) costs more than £60,000 per patient [4].

Although it is a high-profile disease and has attracted a great deal of research attention, the pathogenesis of PD remains poorly understood, with the disease being characterised mainly by its symptoms rather than its cause [6]. Most cases of PD (90%) are idiopathic, with late-onset. This form of PD is believed to be caused by a combination of genetic and environmental factors, with ageing being one of the most significant risk factors [7]. However, over the past 15 years a few Mendelian forms of PD have been discovered, comprising at least 10% of the disease burden. A number of distinct genome regions, known as PARK, have been identified as strongly associated with PD development. Mutations in genes encoding Parkinson juvenile disease protein 2-parkin (*PARK2*), phosphatase and tensin homolog (PTEN)-induced putative kinase 1-​​​​​​​*PINK1* (*PARK6*) and *DJ-1* (*PARK7*) proteins are associated with autosomal recessive forms of PD, presumably by a loss-of-function mechanism, while gain-of-function mutations in genes that encode α-Syn (*PARK1*/4) and leucine-rich repeat kinase 2-*LRRK2* (*PARK8*) are found in autosomal dominant forms [8]. These proteins, in particular PINK1 and parkin, are strongly linked to a pathway that negatively influences the degradation of mitochondria within the cell, known as mitophagy, which is thought to be related to the accumulation of defected mitochondria and dopaminergic neural cell death [9,10]. In this study, the proposed role of disrupted mitophagy in the pathogenesis of PD will be critically reviewed by drawing upon literatures, as well as holistically in the context of Parkinson’s therapy.

Diagnosis

**Figure 2:**
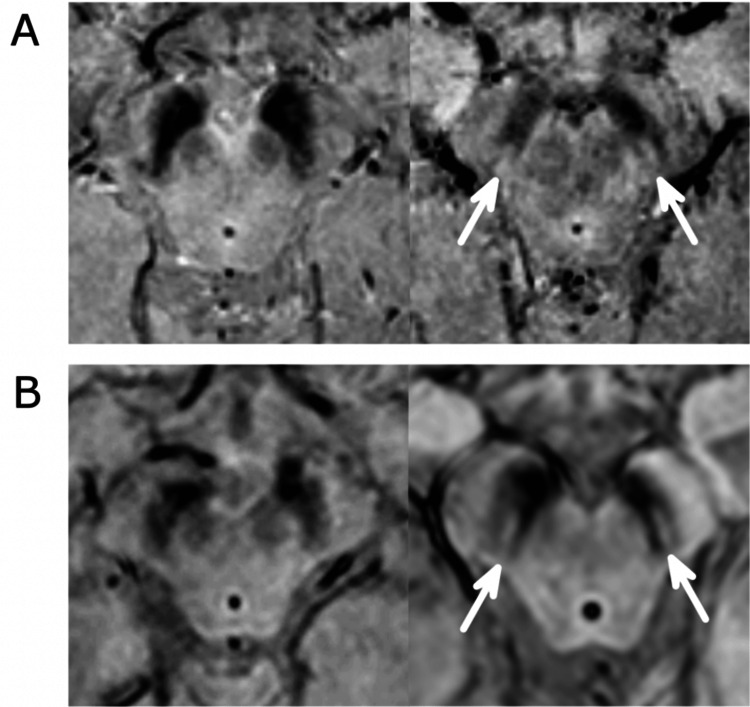
Swallow-tails indicated by arrows (right) present in a healthy female (A) and healthy male (B), or lack thereof in Parkinson’s disease patients (left), female (A) and male (B). Figure taken from [14], and permission was obtained from the original publisher to reproduce the content.

Diagnosis of PD can be challenging as early signs and symptoms may be dismissed as the effects of ageing, causing inaccurate diagnosis. This is complicated as other neurodegenerative disorders such as Creutzfeldt-Jakob disease can produce PD-like symptoms, referred to as Parkinson syndrome. There are, however, studies indicating potential advances in early diagnosis, such as the detection of hypokinetic dysarthria, speech disorders [11], and impaired olfaction, which can occur before motor symptoms develop [12]. Diagnosis may also be confirmed by using single-photon emission computerized tomography (SPECT) or MRI scan when PD cannot be differentiated from essential tremor. For healthy individuals, MRI examination of the substantia nigra will display a “swallow-tail” type appearance. However, such appearance is absent in many cases of PD [13]. These swallow-tails, and the lack thereof, are shown in Figure 2.

In the UK, patients are referred to a specialist and diagnosis is based on the UK Parkinson’s Disease Society Brain Bank Clinical Diagnostic Criteria [15], which looks for the presence of motor symptomatology, particularly bradykinesia, and a number of non-motor symptoms such as dementia and depression.

**Figure 3:**
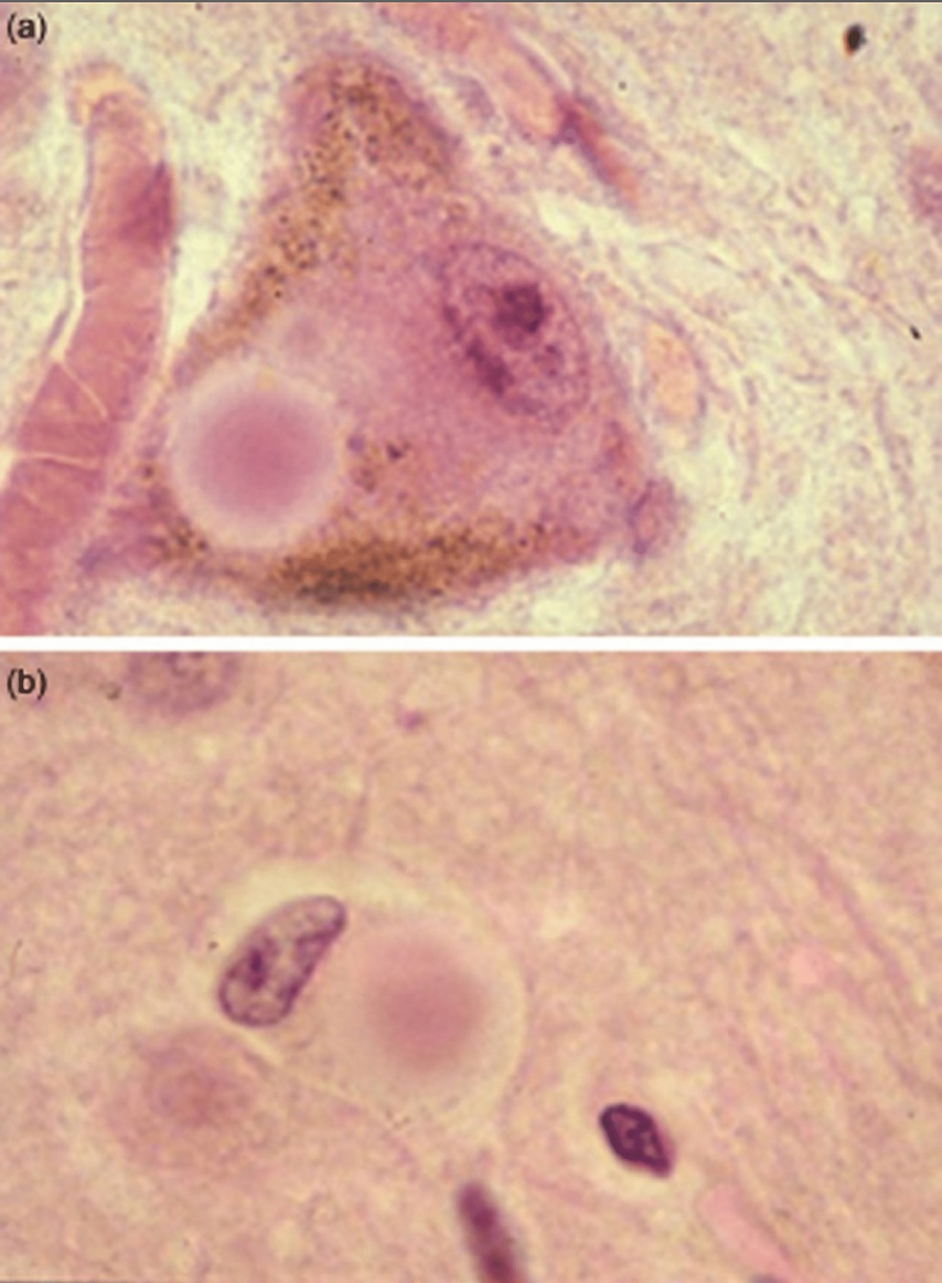
Lewy bodies (pink circular) present in brain tissue. Figure taken from [19], and permission was obtained from the original publisher to reproduce the content.

Post-mortem examination of the brain reveals abnormal accumulation of proteins into globular structures known as Lewy bodies (LBs) within the nerve cells, which can become large enough to displace other cellular components, as illustrated in Figure 3 [16,17]. LBs are composed of a combination of proteins including alpha-synuclein (α-Syn), ubiquitin, neurofilament protein, etc [18,19]. Tau proteins (which stabilise microtubules) and parkin can aggregate with α-Syn in the LBs [20,21]. It is highly likely that the aggregation of the misfolded proteins into LBs is an active process within the cell, with the aim of minimising the damage caused by aberrant proteins by condensing them into a single location rather than allowing their distribution throughout the entire cell. This active formation of LBs would classify as a type of aggresome [22,23]. However, it is this very attempt to prevent widespread cell damage that ultimately induces cell death via the hyperactivation of adenosine monophosphate (AMP)-activated protein kinase [24].

Treatment

PD is currently incurable. Nonetheless, several symptomatic treatments are available with levodopa (L-DOPA) being the primary treatment in early PD [25,26]. L-DOPA is a precursor to dopamine, converted by the enzyme DOPA decarboxylase, and is administered to restore dopamine levels in the brain. L-DOPA is given instead of dopamine as the latter cannot cross the blood-brain barrier, and is often given in combination with a peripheral decarboxylase inhibitor to prevent premature conversion [27,28]. Unfortunately, long-term usage can lead to levodopa-induced dyskinesia as a side effect [29,30]. Various dopamine agonists such as aripiprazole, and MAO-B inhibitors can also be used to alleviate PD symptoms, but with lower efficacy [31]. In advanced PD, the development of motor complications necessitates adjuvant therapy with levodopa and either a Catechol-O-methyl transferase (COMT) inhibitor or dopamine agonist to ameliorate these complications [32]. If patients respond poorly to drug therapy, then surgical options such as deep brain stimulation (DBS) are also available. DBS with an electrode of the subthalamic nucleus and internal globus pallidus is conjectured to control the symptoms by either inhibiting or exciting local neurons [33].

## Review

Disrupted mitophagy in Parkinson’s disease

Natural (Non-pathogenic) Mitophagy Processes

**Figure 4:**
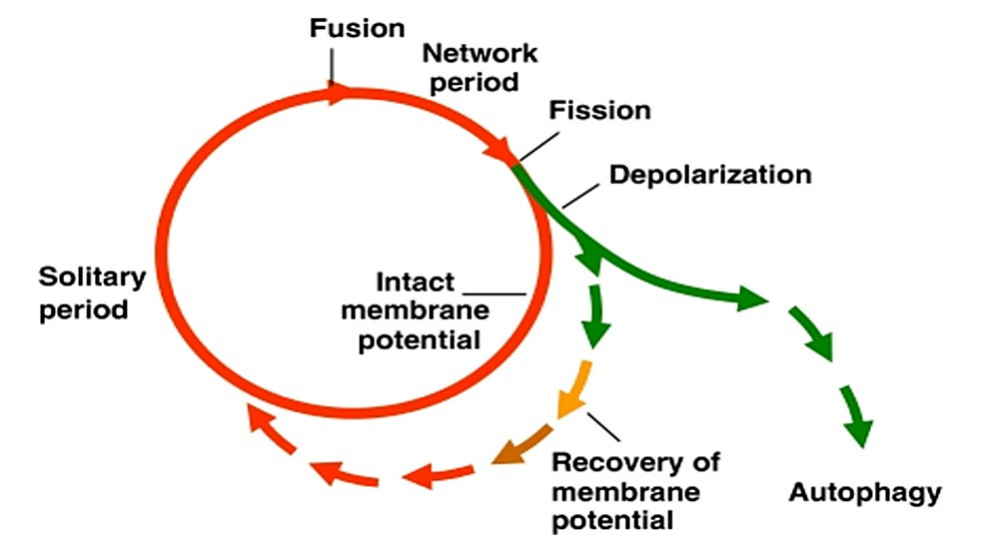
Model of the life cycle of mitochondria. Fusion events are rapidly followed by fission. Daughter mitochondria become solitary for a period before fusing again. Daughters with depolarised membranes may either recover their membrane potential, or are removed by mitophagy. Figure taken from [37], and permission was obtained from the original publisher to reproduce the content.

Mitophagy is defined as the specific and selective, targeted removal of excess or damaged mitochondria from the cell via macroautophagy, a process that utilises autophagosomes to deliver cellular structures to lysosomes for destruction [34]. Dysfunctional mitochondria accumulation as a result of impaired mitophagy has been implicated in several health conditions such as PD, metabolic syndrome, and cancer [35]. Mitophagy plays an important part in mitochondrial proliferation, i.e. enlarging and then a fission event produces two daughter mitochondria. The process is driven by AMP kinase (AMPK) which causes the mitochondria to elongate or fuse and proliferate, producing more ATP and reducing the production of reactive oxygen species (ROS) [36]. If daughter mitochondria have reduced membrane potential, they are selectively removed by mitophagy [37]. Mitochondria are also capable of temporary fusion with each other so that damaged or inefficient mitochondria may briefly receive electrochemical support from a larger mitochondrion, temporarily improving their performance [38,39]. Persistently depolarised mitochondria are targeted for mitophagy as illustrated in Figure 4.

Molecular mechanisms of PINK1/parkin-mediated mitophagy

The processes through which the molecular targeting of dysfunctional mitochondria takes place varies from one species to another, but in humans, the process involves the proteins PINK1, parkin and ubiquitin.

**Figure 5:**
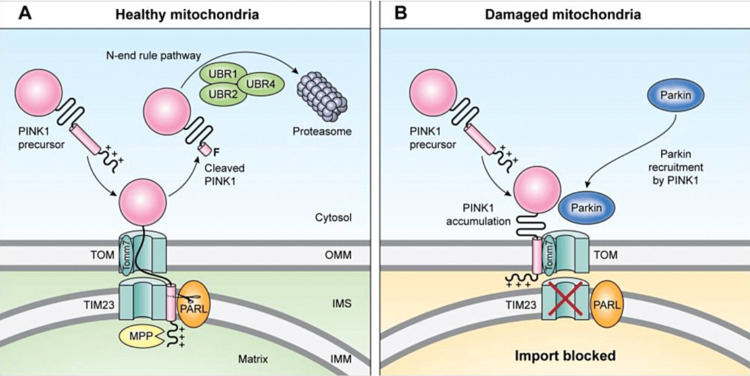
A comparison of healthy mitochondria (A) and damaged mitochondria (B) showing the processing of PINK1 by TOM, TIM, PARL, MPP, and the N-end rule pathway (A), or the accumulation of PINK1 on the OMM and recruitment of parkin (B). Also indicated within the TOM complex is the minor subunit Tomm7, without which the functionality of the entire TOM ceases. PINK1: PTEN-induced kinase 1; Parkin: Parkinson juvenile disease protein 2; UBR1: ubiquitin protein ligase E3 component N-recognin 1; UBR2: ubiquitin protein ligase E3 component N-recognin 2; UBR4: ubiquitin protein ligase E3 component N-recognin 4; TOM: translocase of outer-membrane complex; TIM23: mitochondrial import inner membrane translocase subunit; OMM: outer mitochondrial membrane; IMS: intermembrane space; IMM: inner mitochondrial membrane; Tomm7: translocase of outer mitochondrial membrane 7; MPP: mitochondrial processing peptidase; PARL: presenilin-associated rhomboid-like protein Figure taken from [10], and permission was obtained from the original publisher to reproduce the content.

PINK1, a serine/threonine protein kinase, is a 63 kDa protein found throughout the cytosol and on the outer membranes of mitochondria (OMM), but generally only at low levels due to voltage-dependent proteolysis of PINK1 on healthy mitochondria, which can internalise and degrade PINK1 through a translocase of outer membrane (TOM) complex. The TOM complex then delivers PINK1 to the intermembrane space with the translocase of inner membrane (TIM) complex for cleavage by enzymes including mitochondrial processing peptidase (MPP) and presenilin-associated rhomboid-like protein (PARL) [40]. The resulting PINK1 fragments are then retro-translocated to the cytosol where they can be removed by lysosomal degradation. Due to the primary difference in the cytosolic presentation of PINK1 being an exposed and destabilised N-terminal amino acid, this process is known as the N-end pathway [10,41,42]. Dysfunctional mitochondria, which are not capable of maintaining the necessary inner membrane potential for this removal process, rapidly accumulate large amounts of PINK1 on the OMM which initiates the process of mitophagy [43,44]. This process of mitochondrial labelling is illustrated in Figure 5.

PINK1 is able to recruit parkin from the cytosol, associate and functionally cooperate on the surface of these compromised mitochondria to induce parkin-mediated mitophagy through phosphorylation of both parkin and ubiquitin (both at Ser65), with OMM-bound poly-ubiquitin chains acting as parkin receptors [45-47].

**Figure 6:**
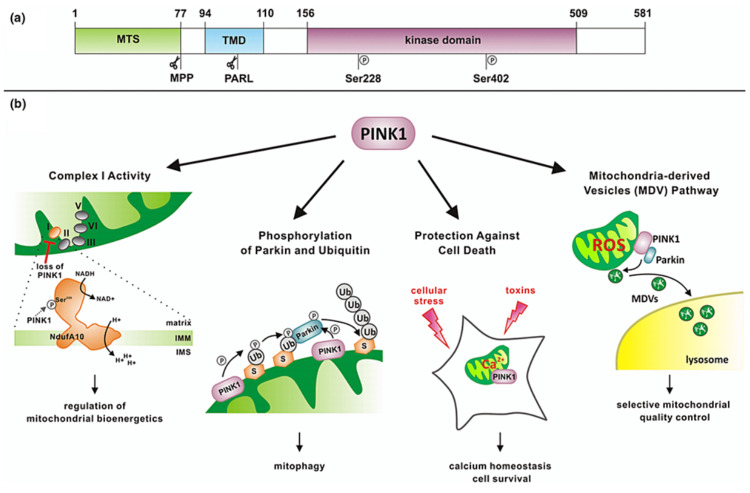
: Domain architecture of PINK1 (a), showing mitochondrial targeting sequence (MTS), the transmembrane domain (TMD), and the kinase domain. Some cellular processes influenced by PINK1 are shown (b) indicating a wide range of areas of activity. MTS: mitochondrial targeting sequence; TMD: transmembrane domain; MPP: mitochondrial processing peptidase; PARL: presenilin-associated rhomboid-like protein; Ser228, Ser402: phosphorylation sites in PINK1; ROS: reactive oxygen species; Ub: ubiquitin; NAD: nicotinamide adenine dinucleotide; NADH: reduced form of NAD; NdufA10: NADH:ubiquinone oxidoreductase subunit A10; IMM: inner mitochondrial membrane; IMS: intermembrane space Figure taken from [52], and permission was obtained from the original publisher to reproduce the content.

Research is ongoing into non-mitophagy-related functions of PINK1, as the protein has been associated with other cell signalling pathways promoting the transport of mitochondria and regulating dendritic morphogenesis [48], along with influencing cell survival under conditions of oxidative stress and regulating complex 1 activity [49,50]. Mutations in the gene coding for PINK1 (*PARK6*) can cause early onset PD while failing to protect dopaminergic neurons from stress-induced mitochondrial dysfunction [51]. Such mutations are associated with increased misfolding of mitochondrial proteins [52]. The involvement of PINK1 in cellular processes is shown in Figure 6.

PINK1 has similarly been associated with mitochondrial trafficking. The OMM-bound GTPases Miro1 and Miro2 are integral to the migration of mitochondria in neurons, through a linking process attaching mitochondria to kinesin and dynein motor proteins [53,54]. Elevated levels of Miro are associated with dopaminergic neurone loss, but the levels can be returned to normal by increasing levels of PINK1. Overexpression of PINK1 was shown to inhibit the movement of mitochondria, resulting in perinuclear clustering [55]. This potentially implicates the Miro proteins and their interplay with PINK1 and parkin in PD pathogenesis.

Parkin is the next step in the PINK1-mediated identification of defective mitochondria. Encoded by the PARK2 gene [56,57], this E3 ligase mediates the deposition of ubiquitin on the surface of target mitochondria through the formation of a multicomponent ubiquitin ligase system which covalently binds ubiquitin to OMM surface proteins [58]. Interaction of parkin with PINK1 induces a change in the functionality of parkin (phosphorylation at Ser65), allowing the sequestration of ubiquitin from the cytoplasm, effectively labelling the dysfunctional mitochondria with a layer of ubiquitin proteins. Parkin is stabilised by neuregulin receptor degradation protein-1 (Nrdp1), which mediates the attachment of ubiquitin to mitochondria-bound parkin [59]. Little is known about Nrdp1 and its role in PD, it may be associated with neuronal apoptosis and protection from ROS [60], but it has been implicated in other areas such as gene expression in pancreatic cancer [61], and immunity [62], among others. PINK1 and parkin are also associated with the processes of mitochondrial fusion and fission, with both proteins having roles in the degradation of excess mitofusins via ubiquitination and subsequent proteasomal degradation, and PINK1 overexpression producing elongated and interconnected mitochondria [9,63]. Parkin has also been demonstrated to have a role in mitochondrial biogenesis through the ubiquitination of a transcriptional repressor called PARIS, which causes its lysosomal degradation. Removal of PARIS results in increased biogenesis of mitochondria [64].

Whilst ubiquitin has several functions related to cell signalling and endocytosis, its relevant action is during the identification of cellular components for destruction [65,66]. First identified in 1975, this ubiquitously expressed 8.5 kDa protein has been identified in plants, yeasts and bacteria, indicating that it is evolutionarily conserved [67]. The ubiquitination of proteins occurs through several mechanisms. Examples of the chemical attachments include ester formation through serine or threonine residues, thioester formation through cysteine and peptide or isopeptide bonds at the N-terminus or lysine residues, respectively [68]. Once the process of PINK1-parkin-dependent ubiquitination has begun, a positive feedback process occurs to strengthen the accumulation of ubiquitin on the mitochondrial surface. Polyubiquitin chains are phosphorylated by PINK1. Phosphorylated polyubiquitin chains have a substantially higher affinity for phosphorylated parkin than for the native form (21 times the affinity) resulting in the recruitment of more active parkin to the mitochondria, its engulfing in an autophagosome, and subsequent delivery for lysosomal breakdown. In summary, ubiquitin is a substrate of parkin, and phosphorylated ubiquitin is an activator of parkin, through binding at an allosteric site. Therefore, ubiquitin is described as a homotropic allosteric regulator, thereby potentiating the effect of the parkin enzyme [10,41,69].

**Figure 7:**
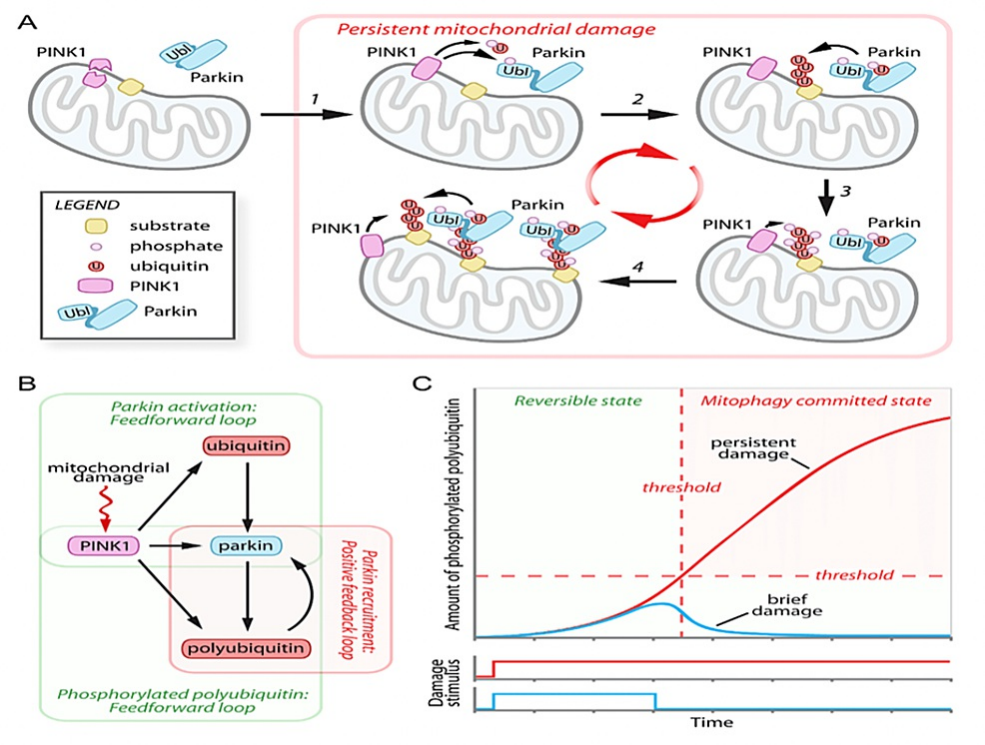
Feedback loops as PINK1, ubiquitin and polyubiquitin enhance the migration of parkin. Which then strengthens the action of PINK1 (A) and (B). Panel (C) shows a hypothetical graph indicating low phosphorylation of ubiquitin where reversal would be possible (blue line) and passing the threshold where damage becomes persistent and the mitochondrion is destroyed (red line). Figure taken from [69], and permission was obtained from the original publisher to reproduce the content.

The feedback loop processes are shown in Figure 7, along with a hypothetical graph which models the levels of damage to targeted mitochondria, showing the point at which the process becomes irreversible.

Mitochondrial recycling through mitophagy in differing cell types

In different cell types, the overall process remains similar, but the specific proteins involved vary from one species to another. In *Saccharomyces cerevisiae* (yeast), genes such as *UTH1*, an OMM protein, act as a marker to signal the mitochondrion to be removed by mitophagy, and knockout studies of this gene showed that in its absence there is a loss of selective mitophagy [70]. Other genes such as *YME1*, which encodes an ATPase, have the opposite effect that leads to an increase in mitophagy after they are knocked out. These findings in yeast demonstrated that mitophagy is closely controlled by a series of interconnected cellular proteins [71]. Recent research suggests that proteins anchored in the inner mitochondrial membrane (IMM) may trigger mitophagy as dysfunctional mitochondria have some compromise in the OMM leading to greater permeability and, therefore, unmasking IMM proteins, which in turn initiate mitophagy [72]. The IMM can become more permeable in the presence of increased levels of free calcium, increased ROS formation and decreased NADPH, resulting in a decrease of electrochemical stability and swelling of the mitochondrion, which leads to mitophagy. However, if mitophagy is impaired, this swelling can lead to OMM rupture and the release of pro-apoptotic proteins [73]. One of the proteins anchored to the IMM is phosphatidylserine decarboxylase, an enzyme that synthesises phosphatidylethanolamine (PE). PE modulates the solubility of α-Syn, and if low levels of PE are present and α-Syn is not controlled, causing mitochondrial defects, along with stress in the endoplasmic reticulum and a 3-fold increase in the levels of α-Syn [74]. This provides a better understanding of the mechanism of the prior observation that mitochondrial defects are associated with the formation of LBs [75].

Autophagy proteins (Atg) have been identified in yeasts as being critical to the mitophagy process, and a mammalian homologue has been found in the form of a Bcl-2-like protein 13 [76]. Atg32 is an OMM-bound 60 kDa protein that confers a level of selectivity to the process of mitophagy [77,78]. Atg32 can bind to Atg11, which then binds to Atg8, or Atg32 can bind directly to Atg8 as the tryptophan (W) and leucine (L) spacing in the binding region of Atg32 is sufficient for either interaction to take place (WXXL, where X represents other amino acids) [79,80]. The mammalian homologue of Atg8 is LC3 (microtubule-associated protein 1A/1B-light chain 3) [81]. Atg8 is bound to isolation membranes, so its interaction with mitochondrial membrane-bound Atg32 is thought to be the trigger for localising mitochondria into autophagosomes [82]. Atg32 is short-lived when expressed on the membrane of healthy mitochondria, and only becomes stable during the mitophagy process when it interacts with Atg11 or Atg8 [83]. While the two proteins are unrelated, this has certain similarity to the membrane instability in PINK1 as described previously, which is only stable on dysfunctional mitochondria.

An assortment of other proteins has involvements in various tissue types, including FUNDC1 (FUN14 domain-containing protein 1), a mammalian mitochondrial outer-membrane protein, which is a receptor for hypoxia-induced mitophagy. It interacts with LC3 via a YXXL binding motif, similar to the above-mentioned Atg32 sequence, but with tyrosine (Y) in place of tryptophan (W) [84]. The serine/threonine protein kinase ULK1 is thought to be involved in mitophagy through its interactions with FUNDC1, through a phosphorylation event [85,86]. Additionally, phosphoglycerate mutase family member 5 (PGAM5), a serine/threonine protein phosphatase, dephosphorylates FUNDC1 when under hypoxic conditions, which enhances its interaction with LC3 [87]. PGAM5 is thought to promote the PINK1/parkin pathway of mitophagy [88].

In the heart, cardiolipin is involved in the mitophagy process, as this IMM phospholipid has not only been detected migrating to the outer mitochondrial membrane and interacting with LC3, but knockout of this protein led to reduced capacity for mitophagy and upregulation of PINK1 in a bone marrow cell line [89].

Sequestosome-1 (ubiquitin-binding protein p62) is one of the most common markers to monitor mitophagy, along with LC3 [90]. Post-mortem analysis has found p62 in several neurodegenerative diseases, and experimentally induced upregulation of p62 has shown disruptions in mitochondrial structure and function [91], despite its known function as a PINK1/parkin-independent mitophagy agent, possibly with a role in immunomodulation [92]. Mutations leading to a failure to express p62, or heterozygous variants, have been associated with amyotrophic lateral sclerosis, Paget disease and frontotemporal dementia [93]. In some cases, healthy mitochondria are targeted for mitophagy. Mammalian red blood cells, for example, do not normally contain mitochondria. During the maturation process of reticulocytes, the OMM protein NIP3-like protein X (NIX) mediates the removal of mitochondria, as NIX-deficient mice have been shown to retain residual mitochondria in their red blood cells [94].

**Figure 8:**
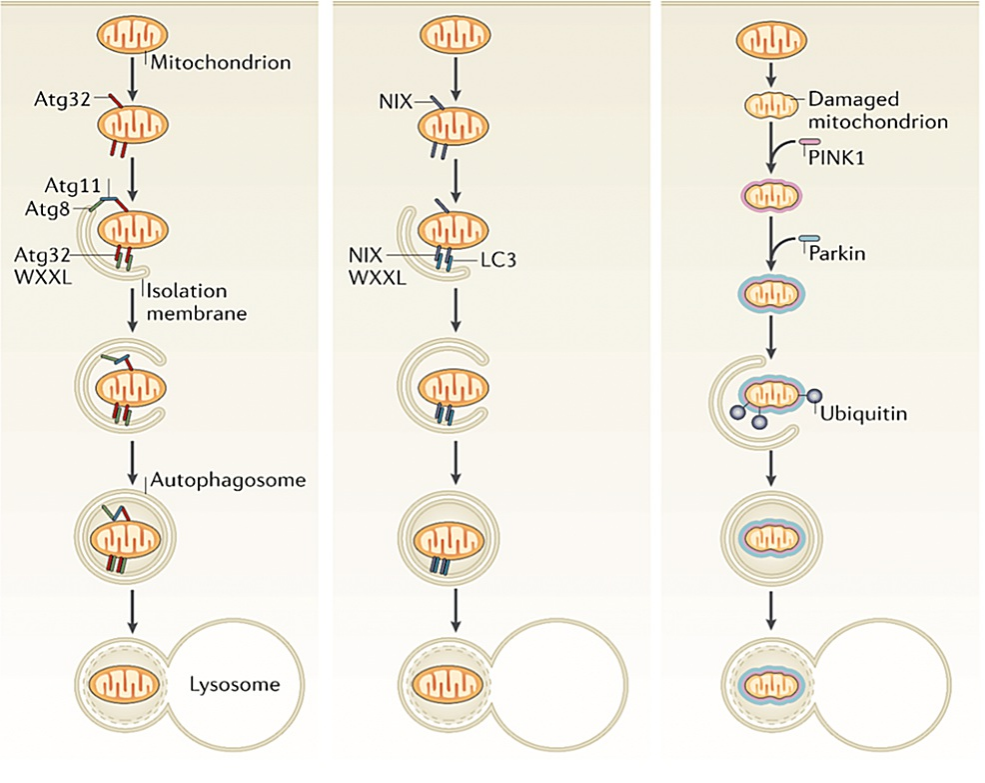
Mitophagy processes in yeast (left), showing Atg32 stabilisation on defective mitochondria and its subsequent interaction with Atg11 and/or Atg8 to associate with an isolation membrane for lysosomal degradation. Red blood cells (centre) dispose of mitochondria through a mechanism thought to involve the membrane protein NIX. PINK1-parkin-induced mitophagy (right). Atg32, Atg11, Atg8: autophagy proteins; WXXL: a conserved amino acid motif associated with LC3; NIX: NIP3-like protein X; LC3: microtubule-associated protein 1A/1B-light chain 3. Figure taken from [82], and permission was obtained from the original publisher to reproduce the content.

The mitophagy processes are complex and whilst the mechanics are gradually being elucidated, the specifics of the signalling stimuli are only partially understood, and significant further research in this area is required [95]. The basic principles and the main differences in the three mitophagy processes mentioned above are illustrated in Figure 8.

In some instances, the targeting of healthy mitochondria may be of benefit. For example, in the ischaemic heart, once reperfusion has commenced, tissue can be damaged by the sudden generation of high levels of ROS, an effect which can be reduced given the removal of mitochondria prior to reperfusion [96]. Ischaemic preconditioning results in the localisation of parkin to mitochondria in isolated perfused rat hearts, with an associated greater cardiomyocyte survival than parkin-knockout mouse hearts [97]. Inhibition of mitophagy with bicarbonate has been shown to increase the risk of reperfusion injury [98]. The localisation of parkin to the mitochondria of cardiac myocytes is independent of PINK1, suggesting that there is another mechanism which could potentially compensate for PINK1 depletion in other cell types [99].

When the process of mitophagy is disrupted, dysfunctional mitochondria are allowed to accumulate in the affected cells. They are less likely to produce ATP and more likely to produce ROS, leading to cell damage or even cell death [100]. Conversely, excessive mitophagy can result in inadvertent apoptosis as the cell no longer has sufficient energy to survive [101,102]. In an effort to prevent excessive mitophagy, the mitochondrial membrane-bound apoptosis-regulating Bcl-2 family of proteins antagonises parkin-mediated mitophagy [103]. During the mitophagy process, Bcl-2 is able to escape from the degrading mitochondria [104]. Altogether, these points indicate the potential for a negative feedback system that would slow the mitophagy process following the accumulation of Bcl-2 in the cytosol after the successful degradation of mitochondria, thereby countering the above-mentioned PINK1-parkin-ubiquitin positive feedback system. Additionally, there are several ubiquitin-specific proteases which are capable of removing ubiquitin chains from parkin targets, thereby directly interfering with parkin-driven mitophagy [105].

As individuals age, the quality control systems that are usually at work within cells ensuring the fidelity of proteins and organelles become gradually compromised [106,107]. Age-related impairment in mitophagy increases the indications of ageing in the heart, such as becoming hypertrophic, fibrotic and increasing muscle stiffness. An accumulation of defective cellular organelles including mitochondria, which gives rise to increased production of ROS, are thought to be responsible for many of these symptoms. If cardiac mitophagy can be restored, it may offer treatments for heart disease.

**Figure 9:**
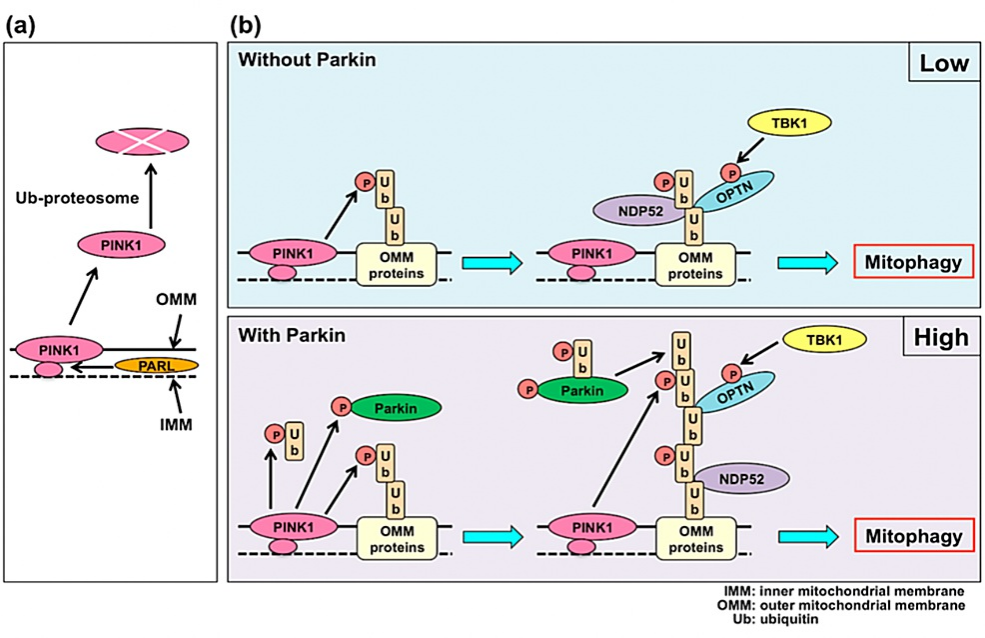
When mitochondria are healthy, PINK1 is degraded (a). When mitochondria are compromised, PINK1 and parkin recruit ubiquitin to label mitochondria for mitophagy (b). PINK1: PTEN-induced kinase 1; PARL: presenilins-associated rhomboid-like protein; OMM: outer mitochondrial membrane; IMM: inner mitochondrial membrane; NDP52: calcium binding and coiled-coil domain 2; OPTN: optineurin; TBK1: TANK-binding kinase 1. Figure taken from [109], and permission was obtained from the original publisher to reproduce the content.

Mitophagy can also be disrupted in humans through genetic mutations which code for any of the proteins involved in this complex process, notably parkin and PINK1, which are known to be mutated in hereditary PD, specifically, mutations which abrogate the function of PINK1 result in early onset PD symptoms [43]. The mitophagy process through activation of PINK1 and parkin is shown in Figure 9, including the engagement of NDP52 and optineurin, parkin-independent mitophagy activators. These two proteins have been shown to be recruited by PINK1 even in the absence of parkin, which go on to recruit other mitophagy factors ULK1, DFCP1 and WIPI1. Together, this study indicates PINK1 accumulation on mitochondria as the trigger for mitophagy, with parkin acting to amplify the signal [108].

Genetics and environmental disruptions to mitophagy in Parkinson’s disease

On the occasions of inherited PD, mutations are identifiable in the genes coding for a range of proteins, including α-Syn, parkin, PINK1, DJ-1, and LRRK2 [110]. An interesting starting point for this analysis is the differences seen in the genetics of PD patients of different ethnicities.

In a study of Czech patients, mutations were found in the gene coding for parkin, PARK2, in around 7% of subjects. Specifically, one patient presented with a point mutation while the others had exon deletions [111]. A similar Polish study identified around 4% of PD patients with mutations in PARK2, but was able to identify a range of heterozygous polymorphisms of PARK2 in 21% of patients [112].

A study that identified the genetic variants of the *LRRK2* gene (*PARK8*) in a Taiwanese population of PD patients found higher expression of one allele (G2385RA) in PD patients in comparison to healthy controls. They also discovered two previously undocumented mutations that may affect the folding structure of *LRRK2* [113]. Genetic studies of Nigerian patients with PD showed no pathogenic mutations when screened for changes in *PARK2* or *LRRK2* [114]. Similarly, a more recent Saudi study seeking mutations in SNCA (coding for α-Syn), *PARK2*, *PINK1*, *PARK7* (*DJ-1*), *LRRK2*, and others, revealed only three mutations (two in *PINK1* and one in *PARK2*) in a study involving 98 patients [115]. They went on to suggest that whilst very little was identified in the way of genetic mutations, gene expression and dosing may be a more pertinent issue, along with the possibility that there are undiscovered gene mutations involved in the development of PD. Mutations in *LRRK2* are a major cause of familial PD and are known to occur in idiopathic PD [116], specifically the G2019S mutation which has been shown to occur in 6% of cases of idiopathic PD in Ashkenazi Jews, and over 29% in familial PD in the same population [117].

Mutations in PINK1 result in an inherited early onset PD [51]. The usual dimerised structure of PINK1 is not affected by mutations of PARK6, but its function is impaired sufficiently to result in deficits in mitochondrial respiration, impairment of proteasome function and increased aggregation of α-Syn [118]. However, mutation of PARK6 does not give rise to a single physiological change. A range of different mutations of PARK6 is possible, and each produces a slightly different pattern of abnormality in the resulting cells. Although, in all cases, reduced mitochondrial membrane potential and greater free radical production are evident [119]. Pesticides such as paraquat can induce parkin aggregation; an effect which is ameliorated by overexpression of LRRK2 [120]. Moreover, the G2019S mutation of LRRK2 has been shown to improve the survival of nematodes exposed to paraquat, which also results in the loss of dopaminergic neurons at an accelerated rate [121]. As dysfunctional LRRK2 is known to be one of the mutations giving rise to PD, this indicates that a combination of genetic and environmental factors may be involved in the onset of PD. Moreover, LRRK2 expression can be altered by mutated PINK1 in the absence of any genetic abnormality in the *PARK8* gene [122].

Mutations in PARK2 give rise to altered parkin protein, which then produces autosomal recessive juvenile parkinsonism [57]. Mutations in parkin in ex vivo cell models resulted in higher respiration rates in mitochondria and greater growth of the cells as a whole, suggesting some kind of compensatory mechanism to preserve mitochondrial function in the absence of parkin [123]. In PD, parkin mutations specifically affecting Ser65 would explain the lack of phosphorylation by PINK1. In an experimental model, a mutated form of parkin (with Ser65 abnormality) is not activated by PINK1, whereas a similar mutation in ubiquitin allowed for ligase activation, demonstrating that phosphorylated ubiquitin was able to activate parkin allosterically [124]. Interestingly, the mitochondrial motility protein Miro1 has altered turnover on damaged mitochondria in PD, with ubiquitination of Miro1 being dependent on this same Ser65 residue within parkin [125].

Errors in these pathways do not only contribute to PD. Cutaneous melanoma occurs more frequently in PD patients than in the general population. A genetic analysis of these patients found a large degree of overlap between mutations in known PARK genes and the development of cutaneous melanoma, suggesting the possibility of a shared pathway in the two conditions [126].

Other possible underlying causes of mitophagy dysfunction

Environmental factors play a role in disrupted mitophagy. Exposure to certain pesticides such as rotenone can be a potential cause of PD, as it can impair mitophagy to damage neurons, but this is rescued by overexpression of DJ-1 [127]. Certain drugs such as amphetamines can induce mitochondrial dysfunction and DNA damage in cultured cells, resulting in increased oxidative stress [128]. Additionally, neurotoxin 1-methyl-4-phenyl-1,2,3,6-tetrahydropyridine (MPTP) can inhibit complex 1 of the mitochondrial respiratory chain and result in increased ROS production and DA degeneration, causing parkinsonism.

**Figure 10:**
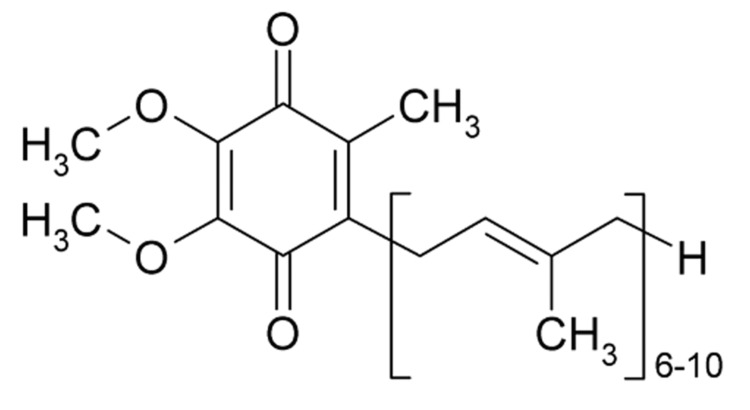
Ubiquinone, also known as coenzyme Q10.

Besides chemical exposure, deficiencies of certain compounds have a role to play in the mitophagy process. Coenzyme Q10, or ubiquinone, due to its ubiquitous presence in most animals, is of fundamental value in the cellular respiration process, forming an integral part of the electron transport chain, and deficiency of it can trigger mitophagy [129,130]. Mitochondrial encephalomyopathy, lactic acidosis and stroke-like episodes (MELAS) is a genetic disorder affecting mitochondrial function and is characterised by muscle pain and weakness, seizures, and symptoms consistent with stroke [131]. In studies involving in vitro models, coenzyme Q10 has been shown to have significantly lower than normal levels, which is accompanied by increased oxidative stress and activation of mitochondrial permeability transition which should bring about mitophagy of the affected mitochondria. However, mitophagy is impaired in these cell models-an observation which is reversed following supplementation with coenzyme Q10 (Figure 10) [132]. Similar findings and benefits of coenzyme Q10 supplementation have been reported for other conditions, such as fibromyalgia [133], heart failure [134] and research is ongoing into the potential role of coenzyme Q10 in “mitochondrial resuscitation” for patients suffering septic shock [135], and as a neuroprotective agent to reduce secondary damage from the surge in ROS resulting from a traumatic brain injury [136].

Coenzyme Q10 has even shown benefits in the management of PD with respect to patients’ ability to carry out “daily activities”, although a meta-analysis on the subject (concluding a benefit in the treatment of PD) was subsequently withdrawn from the Cochrane Library [137]. Patients treated with nicotinamide showed increased mitophagy and the associated decrease in the levels of mitochondria, yet the mitochondria remain to have significantly increased membrane potential and produce ATP at a greater rate than before treatment began [138].

Certain infections have also been shown to influence mitophagy. The hepatitis C virus, for example, once internalised causes the overexpression of PINK1 and parkin and induces perinuclear accumulation of mitochondria and subsequent increased levels of mitophagy [139].

Effect of mitophagy dysfunction within neurons on cell function

Dopaminergic neurons are more sensitive to stresses such as increased ROS than other types of neurons, probably due to their action to oxidise dopamine during its breakdown [105], however, they show a particular sensitivity to reduction in mortalin (mitochondrial 70 kDa heat shock protein-coded by the *HSPA9* gene, and found on mitochondrial membranes or in the endoplasmic reticulum [140]. In mortalin-deficient Drosophila, similar effects are seen in established Drosophila models of PD, such as reduced ATP levels, and shortened life span, which are the result of abnormal mitochondrial function and morphology [141]. Point mutations in mortalin have been implicated in increasing endogenous oxidative stress and decreasing the tolerance of the cells. Mortalin is also known to inactivate p53, thus, preventing apoptosis [142].

Experimental knockout of PINK1 produces loss of the protein throughout the brain, however, the associated breakdown of mitophagy had heterogeneous effects, affecting the midbrain most severely while other areas of the brain seemed more adaptable or more resistant to oxidative stress [143].

When mitophagy is inhibited in mouse midbrains (through cell-specific deletion of Atg7), dendritic ubiquitinated inclusions appear in neurons, which themselves appear dystrophic and low in dopamine. When the experiment was expanded to include the whole brain, there was presynaptic accumulation of α-Syn and LRRK2 proteins, indicating a link between disrupted mitophagy and the physiological effects of PD [144].

Proposed model of mitophagy mechanisms leading to Parkinson’s disease

Impairment of various processes is well-established in ageing cells, e.g. including not only mitochondrial quality control as described previously, but mutations can often develop in other proteins produced by the endoplasmic reticulum. Indeed, impaired autophagy, mitochondrial dysfunction and metabolic distress are associated with cellular ageing [145]. This age-related inaccuracy in production of PINK1 could be potentially ameliorated by functional parkin, because, as described previously, parkin has been shown to induce mitophagy in the absence of PINK1 in some cell types. Errors in the production of parkin, however, could be more problematic. Disruption of both these proteins can interfere not only with mitophagy but also with mitochondrial fission and fusion events, and mitochondrial trafficking within the neuron.

Age-associated dysfunctional mitochondria can lead to increased levels of ROS, which in turn can make the IMM more permeable, causing swelling and further damage. Compromise of the inner membrane could result in increased levels of α-Syn through the action of the inner membrane-bound enzyme phosphatidylserine decarboxylase.

So, dysfunctional mitochondria can lead to overproduction of α-Syn, and its subsequent aggregation into LBs; yet increased levels of α-Syn can cause further disruption in mitochondrial function, creating an amplifying negative cycle [146]. LBs are composed primarily of α-Syn, a small portion of which is ubiquitinated. This ubiquitination may be important in the formation of LBs, possibly providing an aggregation seed [18].

Given larger concentrations of α-Syn in the cell, likely resulting in aggregation into LBs, coupled with the fact that cells deficient in PINK1 and parkin are more sensitive to oxidative stress, it is unsurprising that cellular damage possibly leading to apoptosis can occur in sensitive cells [9]. As described previously, the substantia nigra with its rich dopaminergic neuron population is more sensitive than other parts of the brain. Cell dysfunction in this area gives rise to the characteristic symptoms of parkinsonian syndromes, if not PD itself.

**Figure 11:**
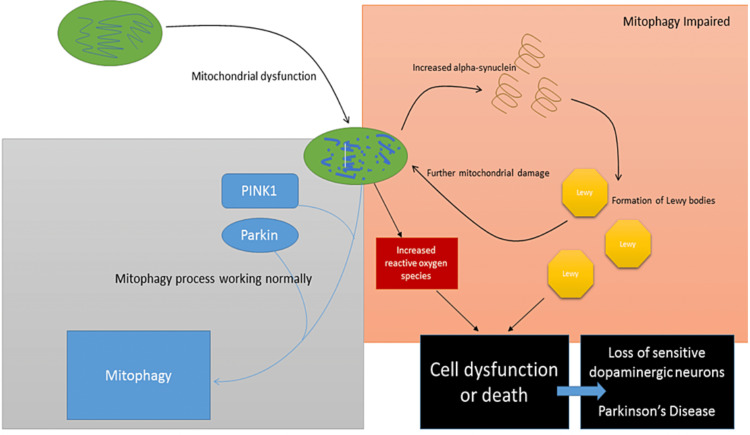
Model of dysfunctional mitophagy leading to increased reactive oxygen species, formation of Lewy bodies, cell dysfunction and death of sensitive dopaminergic neurons—leading to PD. PINK1: PTEN-induced kinase 1.

The model is summarised in Figure 11.

Overall discussion and summary

The model described above lends itself to the pathogenesis of idiopathic PD, however, in familial PD the described process would simply be accelerated independently of the ageing process through mutations in the genes coding for parkin and PINK1. Indeed, these mutations are often associated with early onset variants of the condition (Table 1).

**Table 1:** Genetics of Parkinson's disease. The table contains known PD-related loci and whether mutations result in classical PD or early onset PD. Table taken from [147], and permission was obtained from the original publisher to reproduce the content.

Symbol	Gene locus	Disorder	Inheritance	Gene	Status and remarks	Mode of identification
PARK1	4q21-22	EOPD	AD	SNCA	Confirmed	Linkage analysis
PARK2	6q25.2–q27	EOPD	AR	Parkin	Confirmed	Linkage analysis
PARK4	4q21–q23	EOPD	AD	SNCA	Erroneous locus (identical to PARK1)	Linkage analysis
PARK5	4p13	Classical PD	AD	UCHL1	Unconfirmed (not replicated since described in 1998)	Functional candidate gene approach
PARK6	1p35–p36	EOPD	AR	PINK1	Confirmed	Linkage analysis
PARK7	1p36	EOPD	AR	DJ-1	Confirmed	Linkage analysis
PARK8	12q12	Classical PD	AD	LRRK2	Confirmed; variations in *LRRK2* gene include risk-conferring variants and disease-causing mutations	Linkage analysis
PARK16	1q32	Classical PD	Risk factor	Unknown	Confirmed susceptibility locus	Genome-wide association studies
PARK17	16q11.2	Classical PD	AD	VPS35	Confirmed	Exome sequencing
PARK18	3q27.1	Classical PD	AD	EIF4G1	Unconfirmed; recently published	Linkage analysis

Yet, disrupted mitophagy and genetic predispositions towards it may not be the only issues to consider. Mutations in the protein deglycase DJ-1 (coded for on the *PARK7* gene) are also known to be instrumental in the development of autosomal recessive early onset PD, specifically as its native form increases tolerance to ROS and inhibits the aggregation of α-Syn [148,149]. This appears to be entirely distinct from the mitophagy system, yet still protecting neurons from oxidative damage and subsequent cell death, while also reducing the formation of LBs [150]. Recent research in this area has yielded an antibody to the C-terminus of α-Syn which has been shown to inhibit fibril formation, which may potentially provide benefit in the immunotherapy of PD in years to come [151].

Other factors also come into play in the pathogenesis of PD. For example, the abnormal accumulation of α-Syn may not purely be a result of mitochondrial dysfunction. After the observation that the native enzyme responsible for the degradation of α-Syn (cathepsin D) is upregulated in some PD models, attention has been turned to glycosaminoglycans as regulators of cathepsin D [152]. Indeed, mutated forms of α-Syn can begin to cause PD-like non-motor symptoms in Drosophila prior to the formation of the characteristic fibrils that make up LBs [153].

The connection between disrupted mitophagy and PD has already been reviewed, but the authors quite rightly pointed out both a lack of available evidence in cell or animal models of PD and a lack of known pharmacological mitophagy enhancers, which would obviously be the goal of research aiming towards PD therapy [154]. Having said that, a mitophagy inducer was recently tested in cell culture that was found to successfully engage mitochondrial quality control and autophagy without requiring the recruitment of PINK1 or parkin. This showed none of the negative effects associated with usual experimental mitophagy inducers, however, this research is in its early stages [155]. Additionally, nicotinamide-related enhancement of mitophagy was briefly mentioned previously, with the authors speculating that increased mitophagy was resulting in decreased mitochondrial count, but increased efficiency in each mitochondrion. Further research in this area has allowed the elucidation of the mechanism involved: activation of an NAD-dependent deacetylase called SIRT1 (silent mating type information regulation 2 homolog). SIRT1 activators were subsequently shown to increase mitophagy but did not affect residual mitochondrial efficiency, which was dependent upon the metabolic state of the cell [156].

The established opinion which considers PD as a single condition pertaining to loss of dopaminergic transmission is currently being challenged as there is evidence that non-dopaminergic pathways can also be damaged in the condition. As there is a range of non-motor subtypes (e.g. cognitive disorders), and abnormal accumulations of α-Syn have been observed in the heart, gut, pancreas and skin, the theory has been put forward that PD would be better considered a syndrome rather than a single disease, with multiple dysfunctional neurotransmitter pathways in both central and peripheral regions of the nervous system [157].

Recommendations for future primary and secondary research

A pharmacological regulator of mitophagy is likely the next large step to be taken in the treatment of PD, so research to continue the work of Campanella and East and East et al. is important [155,158].

**Figure 12:**
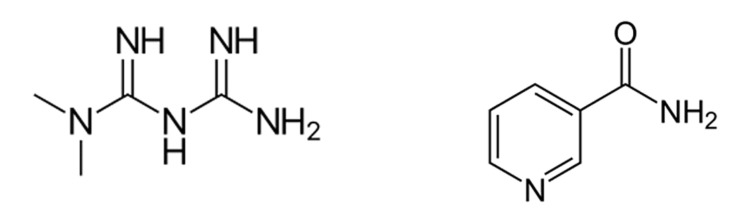
Structures of metformin (left) and nicotinamide (right)—both of which have been associated with the enhancement of mitophagy.

Interestingly, despite wide genetic heterogeneity, patients suffering from diabetes mellitus have a decreased incidence of PD, although the mechanism of this apparent resistance is unclear [159]. One possible research avenue that would be of particular interest is the restoration of dysfunctional parkin-mediated mitophagy that has been seen with the antidiabetic medication metformin [160]. They observed that treatment of mice with metformin resulted in a reduction in stress level in the endoplasmic reticulum combined with decreased expression of p53, a protein known to inhibit the translocation of parkin. Activation of AMPK in hepatocytes has been proposed as a potential mechanism for metformin (Figure 12).

PINK1 and parkin have also been shown to be upregulated in the vasculature of obese and diabetic mice, so research to elucidate the interplay between PINK1/parkin, mitophagy, diabetes and PD may help to clarify our understanding [161].

Potential therapeutic benefits of this research

Research in this area could revolutionise the treatment of PD. Patients who currently understand that the disease is progressive and incurable could be offered mitophagy-enhancing treatments which may confer a neuroprotective effect, thereby slowing disease progression. If the concurrent research into immunotherapy, with antibodies to α-Syn being developed, can be expanded upon, this offers the possibility of a double-edged attack on this devastating neurodegenerative condition.

## Conclusions

Attempts to directly associate mitophagy with PD by discussing alterations in PINK1 or parkin would be a little short-sighted, as both proteins are not only involved in mitophagy but are also active in mitochondrial trafficking and fusion/fission events.

Having said that, there is a strong link between disruptions in mitophagy, the associated accumulation of dysfunctional mitochondria, the formation of LBs, and the destruction of dopaminergic neurons, so the mitophagy process is clearly a central player in PD pathophysiology. Whether particular proteins, or mutations thereof, can be identified as the initiator(s) of such dysfunction is unlikely, but remains to be seen. Rather, the mitophagy process as a whole should be thoroughly researched and understood with a view to the generation of therapeutic agents capable of restoring function, and in doing so, decelerating the destruction of dopaminergic neurons, slowing PD progression.

## Appendices

Atg                  Autophagy protein

α-Syn              Alpha-synuclein

COMT            Catechol-O-methyl transferase

FUNDC1        FUN14 domain-containing protein 1

LBs                  Lewy Bodies

LC3                 Microtubule-associated protein 1A/1B-light chain 3

LRRK2           Leucine-rich repeat kinase 2

OMM              Outer Mitochondrial Membrane

MELAS          Mitochondrial encephalomyopathy, lactic acidosis, and stroke-like episodes

NADP             Nicotinamide adenine dinucleotide phosphate

Nrdp1              Neuregulin receptor degradation protein-1

NIX                 NIP3-like protein X

Parkin              Parkinson juvenile disease protein 2

PD                   Parkinson’s disease

PGAM5          Phosphoglycerate mutase family member 5

PINK1             PTEN-induced putative kinase 1

PTEN              Phosphatase and tensin homolog

ROS                 Reactive Oxygen Species
